# Preparation and Characterization of Azelaic Acid‐Loaded Nanofiber Scaffold as Anti‐Acne and Depigmenting Care, Fabricated by Electrospinning Method

**DOI:** 10.1111/jocd.71202

**Published:** 2026-09-21

**Authors:** Niki Yadegari, Fatemeh Majdi, Nooshafarin Amani, Nasrin Samadi, Farid Abedin Dorkoush, Hamid Akbari Javar

**Affiliations:** ^1^ Department of Pharmaceutics, Faculty of Pharmacy Tehran University of Medical Sciences Tehran Iran; ^2^ Department of Pharmaceutical Biomaterials, Faculty of Pharmacy Tehran University of Medical Sciences Tehran Iran; ^3^ Department of Food and Drug Control, Faculty of Pharmacy Tehran University of Medical Sciences Tehran Iran; ^4^ Pharmaceutical Quality Assurance Research Center, the Institute of Pharmaceutical Sciences (TIPS) Tehran University of Medical Sciences Tehran Iran

**Keywords:** acne, azelaic acid, chitosan, electrospinning, nanofiber

## Abstract

**Objective:**

Acne vulgaris is a common dermatological concern, and conventional topical azelaic acid formulations may have limitations in skin residence time and tolerability. Polymeric nanofiber scaffolds offer a promising platform for topical drug delivery. This study aimed to develop and characterize chitosan/polyvinyl alcohol (CS/PVA) electrospun nanofibers loaded with azelaic acid for potential use in acne management.

**Method:**

Azelaic acid‐loaded CS/PVA nanofibers were prepared by electrospinning. Morphology was evaluated by scanning electron microscopy, in vitro drug release was assessed by an immersion method, antibacterial activity against *Cutibacterium acnes* was determined by disk diffusion, and physicochemical stability was examined under accelerated conditions.

**Results:**

Nanofibers containing 9% PVA, 2% chitosan, and 50 or 70% azelaic acid showed uniform, bead‐free morphology and suitable physical and mechanical properties under optimized electrospinning conditions. The azelaic acid‐loaded scaffolds exhibited antimicrobial activity against *C. acnes*, acceptable stability, and a sustained release profile, with the 70% formulation releasing over approximately 89% of the drug within 24 h.

**Conclusion:**

Azelaic acid‐loaded CS/PVA nanofiber scaffolds demonstrated favorable in vitro physicochemical and antibacterial properties as topical delivery systems. The 70% azelaic acid formulation was selected as the optimized scaffold for further preclinical investigation, while in vivo and clinical studies are needed to evaluate its therapeutic efficacy and safety.

## 1 Introduction

Acne vulgaris is a common chronic inflammatory disease of the pilosebaceous unit, especially in adolescents and young adults. It is a multifactorial disorder in which several pathogenic mechanisms interact, including follicular hyperkeratinization, androgen‐mediated increase in sebum production, alterations in the skin microbiome (particularly *Cutibacterium acnes*), and complex inflammatory and immunological responses. Rather than being caused solely by *C. acnes* or excess sebum, these factors collectively lead to the development of both non‐inflammatory and inflammatory lesions. Owing to its high prevalence, risk of scarring and post‐inflammatory hyperpigmentation, and considerable psychosocial impact, there remains a continuous need for effective and well‐tolerated topical therapies [1, 2, 3].

Additionally, hyperkeratinization of the follicular epithelium leads to the obstruction of sebum flow within the pilosebaceous canal, resulting in the formation of comedones. The excessive sebum accumulation in this canal creates an environment conducive to bacterial colonization by *C. acnes* and *Staphylococcus epidermidis*, which can trigger an inflammatory response through various mediators [3].

The choice of treatment for acne depends on the specific clinical class or type of acne. There are various options available, including topical, systemic, surgical, and photodynamic strategies [3]. For localized acne, antibiotics, acids (such as salicylic acid and azelaic acid), benzoyl peroxide, and retinoids are commonly used. Purulent and inflammatory acne can be effectively treated with antibiotics such as clindamycin and erythromycin [4, 5]. Systemic treatment for acne involves the use of bioactive molecules, such as retinoids, hormones, and antibiotics like doxycycline, minocycline, and erythromycin [4, 6, 7].

Azelaic acid possesses bacteriostatic and antibacterial properties that effectively combat both aerobic and anaerobic microorganisms commonly found in acne‐prone skin. Unlike other topical agents, azelaic acid is well‐tolerated by individuals with sensitive skin. It is commonly used as a monotherapy or in combination with other treatments to manage acneiform eruptions and various hyperpigmentation disorders, including melasma [8, 9, 10].

One potential therapeutic approach for acne treatment is the utilization of nanocarriers. These nanotechnological carriers have the ability to enhance the targeted delivery of various bioactive molecules that combat acne while also minimizing any potential side effects. The biodegradation and controlled release of drugs from these nanocarriers are crucial factors in achieving effective results in anti‐acne treatment [4, 11].

- Polymeric nanofiber scaffolds have emerged as attractive carriers for topical drug delivery because their high surface‐area‐to‐volume ratio, porosity, and tissue‐like architecture enable efficient drug loading and close contact with the skin. Their mesh‐like structure resembles the extracellular matrix. These properties make nanofiber patches ideal for a variety of applications, including acne treatment, wound dressing, and drug delivery [12, 13, 14, 15]. Electrospinning is a simple and versatile technique for producing such nanofibers from a wide range of natural and synthetic polymers, while allowing control over fiber morphology, diameter, and drug release behavior. This method is suitable for incorporating bioactive compounds into topical and biomedical scaffolds. These features make electrospun nanofibers promising carriers for topical delivery of azelaic acid in acne‐related applications [16, 17, 18, 19, 20].

The aim of this research is to prepare a suitable and stable formulation of nanofibers composed of chitosan and polyvinyl alcohol polymers loaded with azelaic acid by electrospinning method to improve acne lesions, and also evaluating the in vitro properties and stability of the prepared formulation, including morphological studies, drug release pattern, mechanical properties, hydrophilicity, and antibacterial properties against *C. acnes*.

## 2 Materials and Method

### 2.1 Materials

Azelaic acid (≥ 99%, Merck, Germany; Cat. No. 820116; CAS 123‐99‐9), Medium molecular weight chitosan (Merck, Germany; Cat. No. 448877; CAS 9012‐76‐4), Polyvinyl alcohol fully hydrolyzed average molecular weight (PVA, fully hydrolyzed, average molecular weight ≈72 000; Merck, Germany; CAS 27360‐07‐2), Dimethylformamide (DMF, Merck, Germany; Cat. No. 822275; CAS 68‐12‐2), Ethanol 96% (Merck), Glutaraldehyde (25% v/v in water; Merck, Germany; CAS 111‐30‐8), Acetic acid glacial (Merck, Germany; Cat. No. 100063; CAS 64‐19‐7), Polysorbate 80 (Tween 80; Merck/Sigma‐Aldrich, Germany; Cat. No. 822187; CAS 9005‐65‐6) were purchased.

### 2.2 Preparation of Drug‐Free Polymers Solution

To prepare the electrospinning solution, the first step is to select a suitable solvent for the polymers. Since chitosan polymer is not soluble in water, a 2% acetic acid solution is used. Additionally, azelaic acid is only slightly soluble in water and, due to its lipophilic properties, 25% dimethylformamide (DMF) and ethanol were used as co‐solvents in this research to improve its solubility. The next step is to optimize the ratio of polymers. In order to do so, 9% of polyvinyl alcohol and 2% of chitosan were added to the solution. To prepare the solution of polymers without the drug, the solvent and co‐solvent were first mixed in a glass vial with a specific ratio. Then, the desired amount of polyvinyl alcohol was added to the solvent, the vial was closed, and placed on a heater at 60°C with a magnet rotation speed of 350 rpm for 3 h. Once the polyvinyl alcohol was completely dissolved and a clear and homogeneous solution was obtained, the specified amount of chitosan was added to the solution and stirred at a rotation speed of 350 rpm for 24 h at room temperature. After 24 h, the electrospinning process was performed on the final clear and homogeneous solution using a 5 cc syringe [21].

### 2.3 Optimization of Polymer Solution Without Drugs

Considering that the morphology and quality of nanofibers are primarily influenced by the type and proportion of polymers, with the amount of drug having a lesser impact, we first created polymer solutions with varying amounts and ratios in order to obtain an ideal nanofiber. Once we determined the optimal amounts of each polymer, we repeated the process with the same quantities, this time incorporating the drug [22] and analyzing the results.

### 2.4 The Amount of Azelaic Acid

To determine the appropriate dosage of azelaic acid, the minimum inhibitory concentration (MIC) test was used, as reported in the articles. The initial dose of 200 mg/mL azelaic acid in 96% ethanol was prepared in sterile conditions and then diluted to various concentrations up to 1.56 mg/mL using the serial dilution method. Bacterial growth was tested by pouring 25 mL of Brain Heart Infusion (BHI) agar medium into 10 cm plates and culturing *C. acnes* bacteria using a loop. The plates were then placed in a candle jar to harvest fresh bacteria and transferred to an anaerobic environment using the Anoxomat device, and incubated at 37°C for 48 h. After 48 h, a suspension of the desired bacteria was prepared in physiological serum with an optical density (OD) of 0.3–0.25. A sterile swab was then used to inoculate the bacteria onto the inner walls of the tube, and the excess suspension was removed. The bacteria were then cultured on BHI agar medium in all directions until the plate surface was completely inoculated. Wells with a diameter of 5 mm were created for each concentration and filled with a specific amount of the drug solution. To test the antibacterial properties of the solvent (ethanol), a well was also created in the center of each plate. The plates were then placed back in the candle jar and transferred to an anaerobic environment using the Anoxomat device, and incubated at 37°C for 48 h. The diameter of the inhibition zone was then measured for the mentioned bacteria [23, 24].

### 2.5 Preparation of Solution Containing Polymer and Drug

First, the solvent was prepared in a glass vial using the specified ratios. Next, the desired amount of polyvinyl alcohol was added to the solvent, and the vial was sealed. The vial was then placed on a heater set to 60°C with a magnet rotation speed of 350 rpm for 3 h. Once the polyvinyl alcohol was completely dissolved and a clear, homogeneous solution was obtained, the specified amount of chitosan was added, and the solution was stirred at room temperature for 2 h. After this, the azelaic acid was added, and the solution was left on the stirrer for 24 h under the same temperature and magnet rotation conditions. After 24 h, the final clear and homogeneous solution was used for the electrospinning process using a 5 cc syringe [25].

### 2.6 Optimization of Electrospinning Parameters

The prepared solutions were electrospun under different voltages, which were 18 kV, 20 kV, and 22 kV. The diameter of the needle tip placed on the syringes was 18 G. The tip‐collector distance (TCD) was set in the range of 10–20 cm. The spinning solution flow rate was between 0.5 mL/h and 0.8 mL/h. In the optimization phase, nanofibers were collected on a glass slide and their morphological quality was observed using an optical microscope with a 40× magnification. A suitable nanofiber should be free of droplets and beads, and the fibers should be elongated with a smooth surface [26].

### 2.7 Cross‐Linking of Nanofibers

Due to the high level of hydrophilicity of polymers, it was necessary to create appropriate transverse connections to increase the resistance of produced nanofibers against moisture. In order to do this, glutaraldehyde, which is a widely used stabilizer in tissue engineering, was used. Despite the skin and lung toxicity as well as the severe sensitizing effect of this substance, studies have shown that if cross‐linking is done by its vapors at low temperatures and for a short period of time, unlike the immersion method, the toxicity is very low and negligible [18, 19, 20].

**FIGURE 1:**
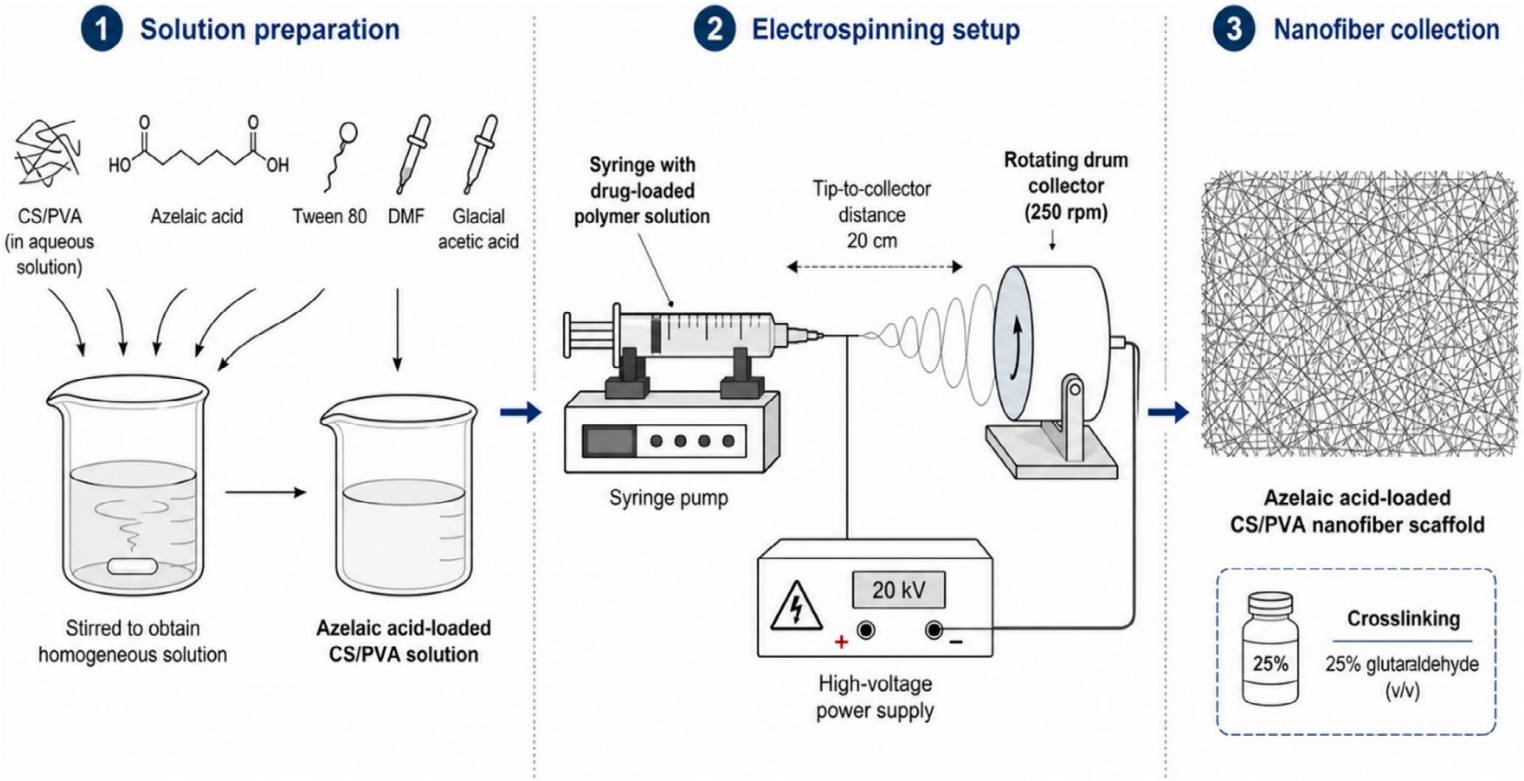
Schematic illustration of the preparation of azelaic acid‐loaded CS/PVA nanofiber scaffolds by electrospinning.

Therefore, in order to create cross‐links, glutaraldehyde solution of 25% in 100% acetone and 35% hydrochloric acid with specified ratios of 75% and 0.01% was prepared for glutaraldehyde activation, and the nanofibers were exposed to the solution vapors for 2 h at a temperature of 55°C in a special chamber. After 2 h, the desired nanofiber was removed from the chamber and kept at room temperature to remove excess glutaraldehyde vapors. Finally, these nanofibers were used in various experiments to determine their properties. Also, a schematic illustration of the preparation of azelaic acid‐loaded CS/PVA nanofiber scaffolds by electrospinning is shown in Figure 1. In this process, the hydroxyl groups present in the structure of polyvinyl alcohol react with the aldehyde groups in glutaraldehyde, and with the exit of the H_2_O molecule, an ether bond is created [27, 28, 29].

### 2.8 Morphological Studies

The surface morphology of the nanofiber scaffolds was examined by scanning electron microscopy (SEM; Seron Technology Co., South Korea). Rectangular pieces of the dried nanofiber mats were cut and mounted on aluminum stubs using double‐sided carbon tape. Prior to imaging, the samples were sputter‐coated with a thin layer of gold (approximately 5–10 nm) under vacuum to improve surface conductivity. SEM images were acquired at an accelerating voltage of 10–15 kV, a working distance of approximately 8–10 mm, and at various magnifications to visualize fiber morphology. Fiber diameters were measured from SEM micrographs using ImageJ software (NIH, Bethesda, MD, USA) by randomly selecting at least 50 fibers per sample [30, 31].

### 2.9 FTIR‐ATR Analysis

FTIR spectra of raw materials samples and nanofibers containing azelaic acid were obtained before and after cross‐linking. In this analysis, by comparing the spectrum of nanofibers with and without transverse connections, the effect of this operation on changing the structure and functional groups of the materials used in the production of nanofibers was investigated. Bruker's 55 Equinox device was utilized for this analysis at the wavelength of 600 cm^−1^–4000 cm^−1^. The obtained results were analyzed to study the effect of transverse connections on the functional groups of nanofibers [32].

### 2.10 Tensile Strength

In this study, Instron universal testing model 5566 made in England was used. First, the nanofibers prepared in equal sizes were cut into a rectangular shape with dimensions of 0.5 × 3 cm and placed in the jaws of the device. The jaws are slowly and forcefully pulled apart until rupture occurs. In this test, the geometric arrangement and interaction between nanofibers determine the properties of the nanofiber network [33].

### 2.11 Contact Angle

The surface hydrophilicity and hydrophobicity of prepared nanofibers can be investigated by the contact angle test. The nanofiber network was cut into smaller pieces with the size of 1 cm^2^ and placed on a surface to determine its contact angle with a drop of distilled water. A syringe filled with 2 mL of distilled water was located perpendicular to the nanofiber surface. A drop of distilled water with a volume of about 15 μL was poured on the grid. The image of the drop was taken with Veho vms‐004 digital microscope and processed by ImageJ software [34].

### 2.12 Swelling Test

After cross‐linking, the cross‐linked samples were analyzed for water absorption. They included drug‐free nanofibers with 50% and 70% azelaic acid. Each of the nanofibers was placed in 5 cc of saline phosphate buffer with pH 4.7, in a sealed vial and in an incubator with a temperature of 37°C, and their weights at 0.5, 1, 2, 4, 6, 8, 12, and 24 h were measured. The weight variations percentage and the amount of water absorption were calculated by Formula 1 [35].

$$\text{Percentage of weight gain}=\frac{\text{secondary weight}\;\mathrm{at}\;a\;\text{specified time}-\text{Primary weight}}{\text{Primary weight}}$$

Formula (1): Calculation of weight variations percentage and amount of water absorption in swelling test.

### 2.13 In Vitro Release of Azelaic Acid From Nanofibers

A calibration curve for azelaic acid was first established by UV–visible spectrophotometry. Briefly, 50 mg of azelaic acid powder was dissolved in 100 mL of phosphate buffer to obtain a stock solution, from which a series of standard dilutions was prepared. The absorbance of each standard solution was measured at 203.8 nm, and a linear standard curve was constructed to correlate absorbance with azelaic acid concentration [36].

The in vitro release of azelaic acid from the nanofibers was then investigated using an immersion method. Approximately 10 mg of each nanofiber sample was placed in a dialysis bag (molecular weight cut‐off: 12 kDa), and 1 mL of phosphate buffer was added inside the bag. The dialysis bag was transferred into a vial containing 10 mL of phosphate buffer as the receptor medium and incubated in a shaker at 37°C. At predetermined time intervals, 1 mL of the receptor medium was withdrawn and replaced with an equal volume of fresh buffer. Drug release studies were performed at neutral pH, and the azelaic acid concentration in the collected samples was quantified at 203.8 nm using the previously established calibration curve [37, 38].

### 2.14 Mathematical Modeling of In Vitro Azelaic Acid Release Data From Nanofibers

Drug release mechanism for nanofibers containing 50% and 70% Azelaic acid can be predicted by mathematical models as general guidelines. This can be achieved by matching in vitro Azelaic acid release (cumulative data) to various models including zero order (Equation 1), first order (Equation 2), Higuchi (Equation 3), and Korsmeyer‐peppas (Equation 4) kinetic models.

(1) Mt=K0t

(2) LogMt=logM0–K1t/2.303

(3) $$\mathrm{Mt}/M\infty ={\mathrm{KHt}}^{1/2}$$

(4) $$\mathrm{Mt}/M\infty =K^{'}\;t^{n}+b$$

M0 and Mt. Represent the initial amount of drug released and the amount of drug released at time t, respectively. Mt/M∞ is the fraction of drug released in time t. K0, K1, KH, and K′ are zero order, first order, Higuchi, and Korsmeyer‐peppas models rate constants, respectively. “n” is release exponent and “b” is related to burst effect [39].

### 2.15 Antibacterial Test

The antibacterial properties of the prepared nanofibers were investigated by determining the diameter of the inhibition zone or disc diffusion against *C. acnes*. To cultivate the bacteria, 25 mL of BHI agar medium was sterilized using an autoclave and poured into 10 cm plates. The bacteria were then cultured on the plates using a loop. To ensure the bacteria were fresh, the plates were placed in a candle jar and then transferred to an anaerobic environment using an Anoxomat device and incubated at 37°C for 48 h. After 48 h, a suspension containing the desired bacteria was prepared in physiological serum with an optical density of 0.3–0.25. To inoculate the bacteria, a sterile swab was dipped into the suspension and then gently touched to the inner walls of the tube to remove any excess. The bacteria were then evenly spread on the BHI agar medium by swabbing in all directions until the entire plate surface was covered.

Then, the prepared drug‐free nanofiber samples containing 50% azelaic acid and 70% azelaic acid were placed on the plates after making transverse connections that were sterilized for 20 min via ultraviolet rays, and after strengthening the junction of the nanofibers, the plates were placed in the candle jar and placed in anaerobic conditions by the Anoxomat device and in an incubator at 37°C for 48 h. After the incubation process, the surrounding area of each sample was examined in terms of creating inhibition zone diameter [40].

A direct comparison with free azelaic acid under the same assay conditions was not included in the present study; therefore, the specific contribution of the nanofiber scaffold to antibacterial enhancement cannot be conclusively determined and should be addressed in future work.

### 2.16 Determining the Stability of Nanofibers Containing Azelaic Acid

The physicochemical stability of the prepared nanofiber was assessed over a period of 12 weeks, with evaluations conducted every 2 weeks. Initially, the drug‐containing nanofiber was stored at a temperature of 45°C. During each evaluation, changes in the organoleptic properties of the nanofiber were monitored as an indicator of its physical stability, while changes in the amount of drug loaded in the nanofiber served as an indicator of its chemical stability. To measure the amount of drug loaded in the nanofiber, 10 mg of the prepared nanofiber was immersed in 5 mL of 96% ethanol for 1 h. It should be noted that polyvinyl alcohol and chitosan are insoluble in ethanol, while azelaic acid has a high solubility in this solvent. Therefore, the drug loaded in the nanofiber was released into the ethanol, and its optical absorption was measured using a UV spectrophotometer. The amount of drug in the solvent was then calculated using a standard curve for azelaic acid [41, 42].

### 2.17 Statistical Analysis

Statistical analysis was performed using SPSS software (version X.X). The normality of data distribution was checked using the Shapiro–Wilk test. Independent *t*‐tests were applied for two‐group comparisons (fiber diameter and drug release). For multiple group comparisons (mechanical properties, hydrophobicity, and antibacterial activity), one‐way ANOVA followed by Tukey's post hoc test was used. Results were reported as mean ± standard deviation (SD), and statistical significance was set at *p* < 0.05.

## 3 Results

### 3.1 Optimization of the Drug‐Free Polymer Solution

**FIGURE 2:**
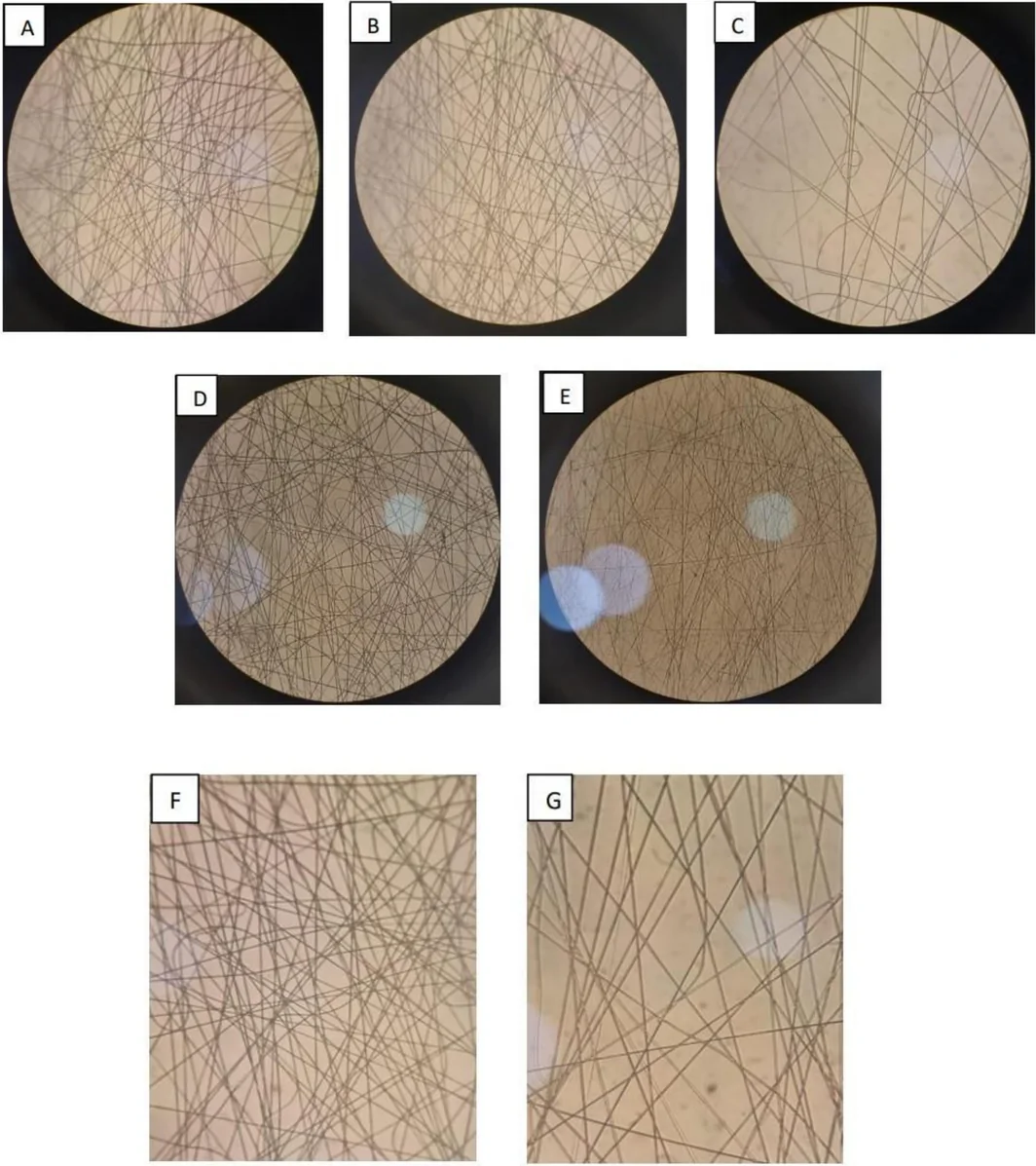
Optical microscopy images of nanofiber scaffolds with different parameters and ratios (40× magnification).

According to Table 1 and Figure 2, which show the results of imaging nanofibers under an optical microscope with a 40× magnification using different parameters and ratios, the most optimal state of the nanofiber was selected. This state was free from droplets and beads and had a smooth surface. These parameters were then used to produce the final formulation. The specific parameters used were as follows:

**TABLE 1:** Optimization of electrospinning parameters and polymer ratio.

Formulation code	PVA%	CS%	AzA%	Voltage (kV)	Flow rate (mL/h)	Needle size (G)	Tip‐to‐collector distance (cm)
A	9	1	0	18	0.5	18	10
B	9	1	0	20	0.5	18	15
C	9	2	0	18	0.8	18	15
D	7	2	0	20	0.5	18	12
E	9	2	0	20	0.8	18	15

### 3.2 The Amount of Azelaic Acid (MIC Test)

**FIGURE 3:**
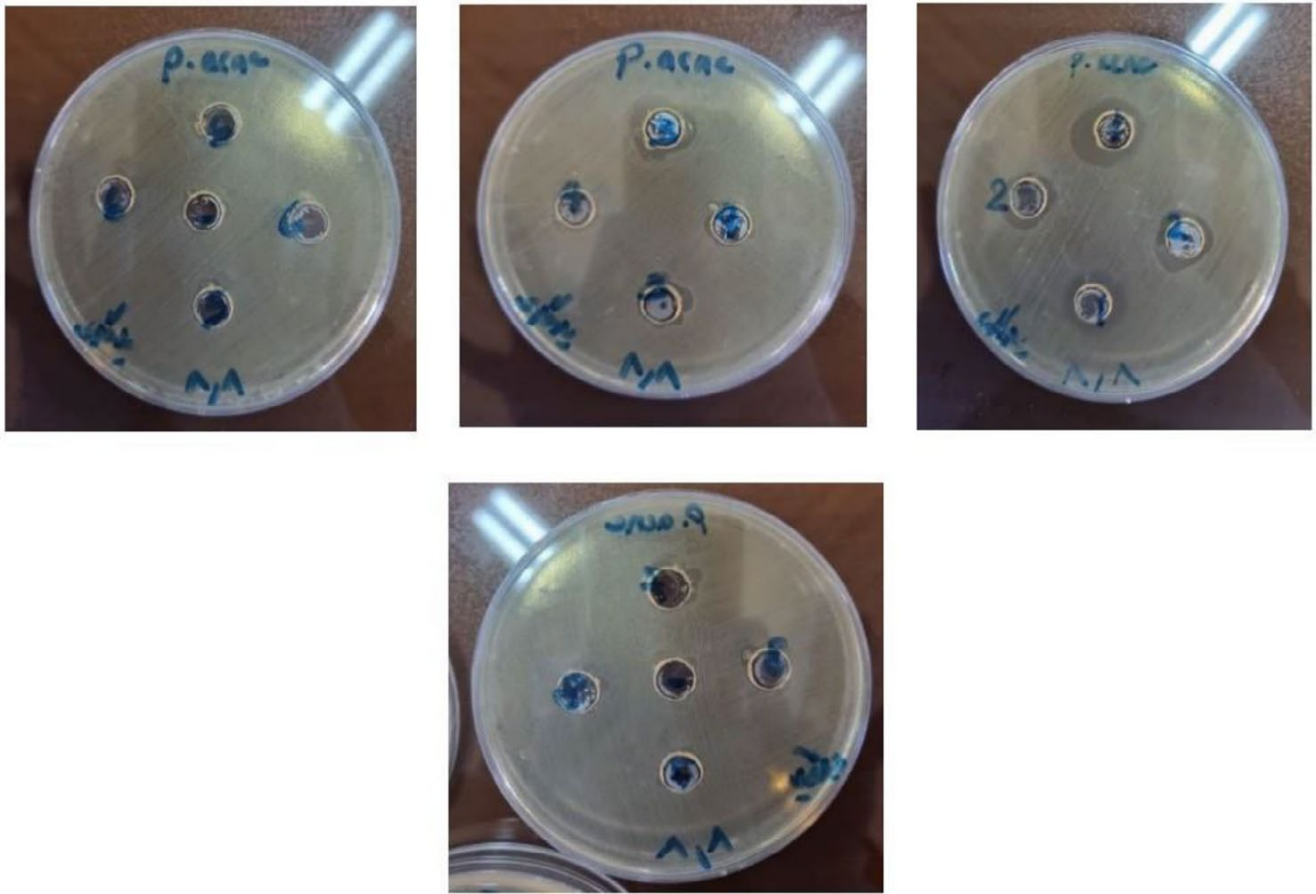
Representative plates of antibacterial assay: Inhibition zones of *C. acnes* at different azelaic acid solution concentrations.

The results related to the minimum inhibitory concentration test were photographed after 48 h of placing the samples in a 37°C incubator, and the inhibition zone for *C. acnes* was observed as in Figure 3 and recorded in Table 2.

**TABLE 2:** Inhibition zones of *C. acnes* at different concentrations of azelaic acid solution (MIC = 25 mg/mL).

Sample code	Azelaic acid concentration (mg/mL)	Inhibition zone diameter against *C. acnes* (mm)
1	200	18
2	100	15
3	50	13.5
4	25	12
5	12.5	No inhibition zone
6	6.25	No inhibition zone
7	3.125	No inhibition zone
8	1.56	No inhibition zone
Blank	Ethanol 96%	No inhibition zone

### 3.3 Optimization of the Drug‐Loaded Polymer Solution

Loading the drug in its minimum inhibitory concentration had a very small effect on the produced nanofibers, and according to this phenomenon, most of the optimized parameters were selected according to the parameters of the polymer solution without the drug, and only the tip‐collector distance (TCD) was slightly increased. Table 3 demonstrates these parameters.

**TABLE 3:** Optimization of electrospinning parameters and polymer ratio for drug‐loaded polymer solution (no significant changes were observed compared to drug‐free polymer solution).

Formulation code	PVA (%)	CS (%)	AzA (%)	Voltage (kV)	Flow rate (mL/h)	Needle size (G)	Tip‐to‐collector distance (cm)
F	9	2	50	20	0.8	18	15
G	9	2	70	20	0.8	18	20

### 3.4 Morphological Results of Nanofibers (SEM)

SEM or scanning electron microscope is a member of the family of electron microscopes that is used to take images of the sample and determine its surface characteristics and morphology. In fact, in this method, the image is recorded by electrons with magnification power of much higher than optical microscopes. These microscopes can take images up to several hundred thousand times. Therefore, in order to investigate the morphology, SEM was used to observe the structure of nanofibers and their distribution diagrams were also drawn using ImageJ software.

**FIGURE 4:**
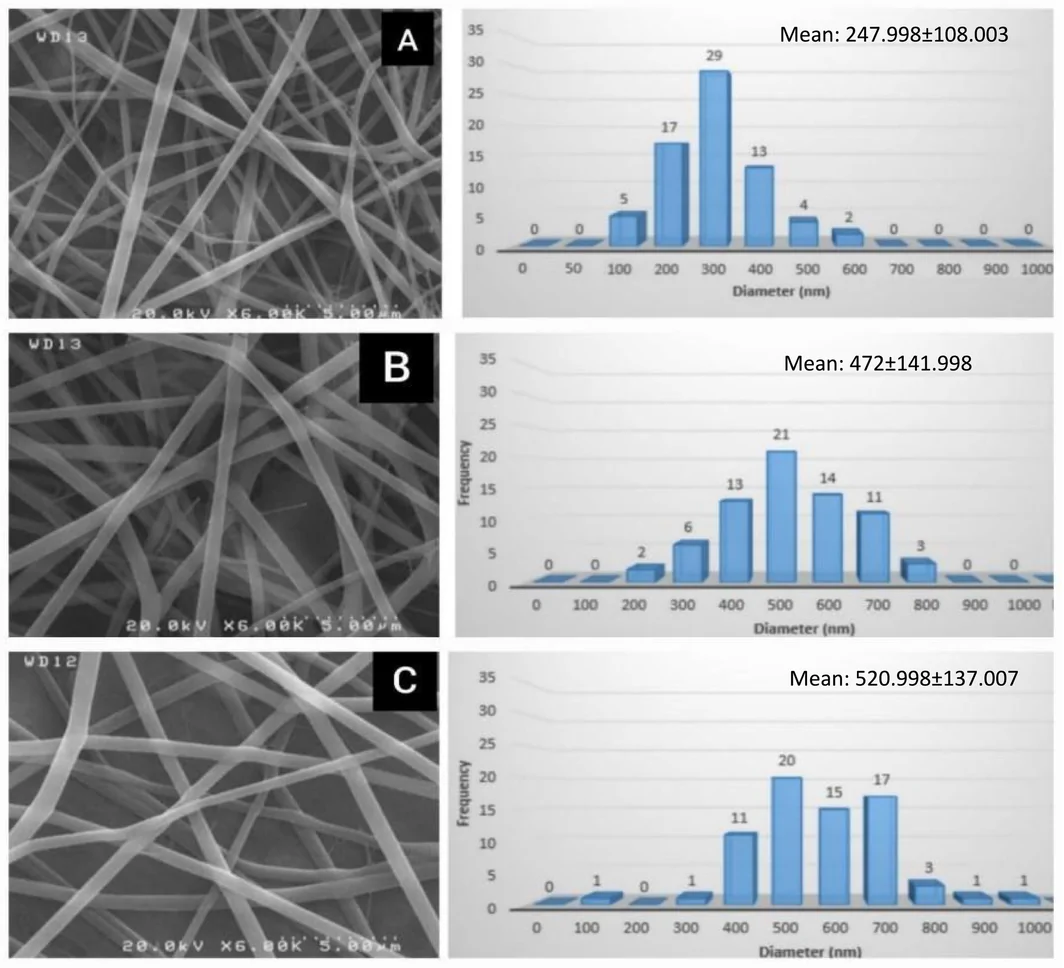
SEM morphology and fiber diameter distribution of CS/PVA nanofibers. (A) Blank CS/PVA nanofibers. (B) CS/PVA nanofibers containing 50% azelaic acid (AzA 50%). (C) CS/PVA nanofibers containing 70% azelaic acid (AzA 70%).

These images are shown in Figure 4. As it can be seen in the pictures, the prepared nanofibers in each sample have a uniform structure without beads. Picture A shows PVA/CS nanofibers, and picture B shows PVA/CS nanofibers containing 50% azelaic acid, and picture C shows nanofibers containing 70% azelaic acid. By comparing the average diameter of nanofibers, we came to the conclusion that by adding azelaic acid, the diameter of nanofibers increases slightly. The average diameter of nanofibers of all three samples is summarized in Table 4.

**TABLE 4:** Mean fiber diameter of blank and azelaic acid‑loaded CS/PVA nanofibers based on SEM analysis (mean ± SD).

Type of nanofiber	Fiber diameter (nm), mean ± SD
CS/PVA	248 ± 108
CS/PVA + AzA 50%	472 ± 142
CS/PVA + AzA 70%	521 ± 137

### 3.5 FTIR‐ATR Test

**FIGURE 5:**
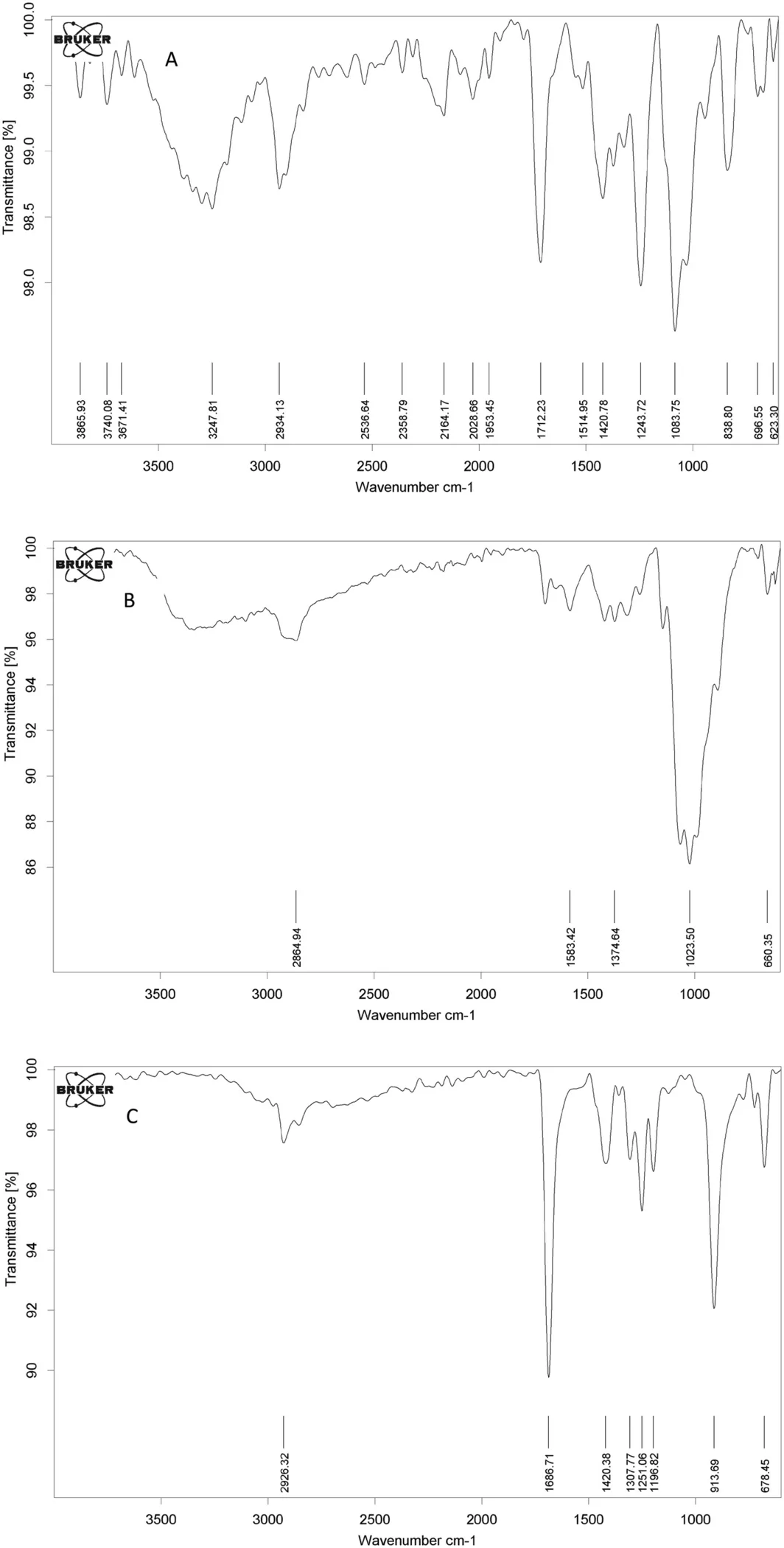
ATR‐FTIR spectra of raw materials. (A) PVA; (B) Chitosan; (C) Azelaic acid.

**FIGURE 6:**
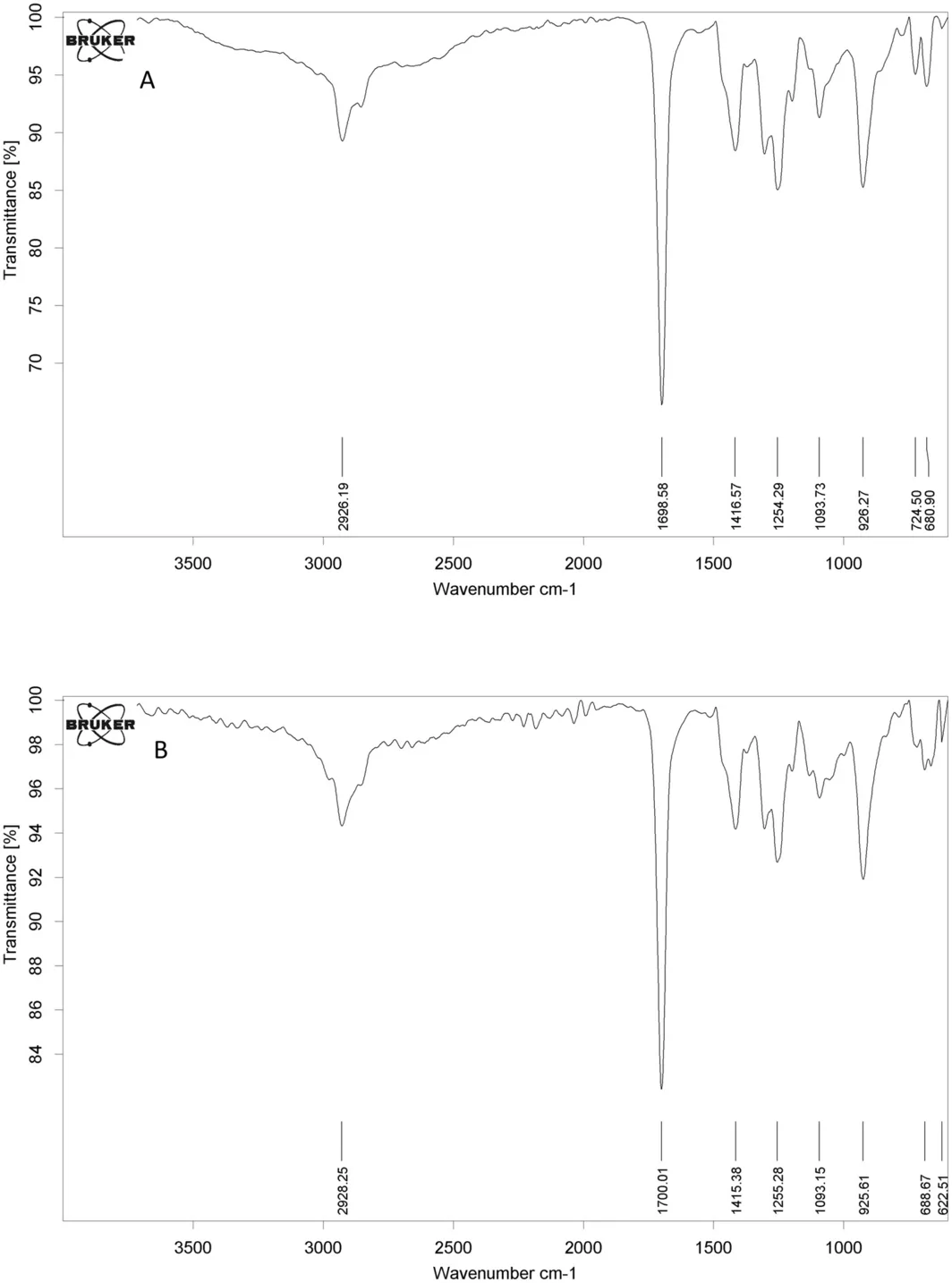
ATR‐FTIR spectra of azelaic acid‐loaded nanofiber scaffolds. (A) Nanofibers containing 70% azelaic acid before crosslinking. (B) Nanofibers containing 70% azelaic acid after crosslinking.

Firstly, the spectrum obtained from polyvinyl alcohol powder was described and examined and it can be seen in Figure 5. In the spectrum related to polyvinyl alcohol powder, a broad peak is observed in the range of 3300–3550 cm^−1^, which indicates the stretching of the O=H bond. Also, the presence of a long peak in the range of 2800–2950 cm^−1^ indicates the stretching of the alkane C=H bond, and the peak around 1085–1120 cm^−1^ also explains the stretching of the C=O bond. A long peak is also seen in the region of 1735–1750 cm^−1^, which is due to the presence of the C=O ester bond. This bond is usually formed between the chains of polyvinyl alcohol by creating an acetate group (Figure 6).

The next spectrum we examined was the spectrum obtained from chitosan powder (Figure 5).

In this spectrum, the peak in the range of 3500–3200 cm^−1^ indicates the presence of O=H bond stretching in the entire structure and also indicates the presence of N=H bond stretching in the N‐Acetyl Glucosamine part of the chitosan structure. The C=N bond in this functional group can be inferred by the 1000–1500 cm^−1^ peak.

The next spectrum we examined was the spectrum obtained from azelaic acid (Figure 5).

In this spectrum, the peak in the 1686 cm^−1^ region shows the stretching of dicarboxylic acid bonds and the peak in 2900–3000 cm^−1^ shows the stretching of the C=H bond of the alkane. Also, the peak in 913 cm^−1^ shows the O=H bond in the carboxylic acid structure.

After examining the spectra related to the raw materials, we evaluated the spectra obtained from the manufactured nanofibers; first, we check the spectrum related to the nanofiber containing 70% azelaic acid before establishing cross‐links, which can be seen in Figure 6.

In this spectrum, the peak in 1250–1300 cm^−1^ explains the stretching of the C=N bond in the amine structure present in chitosan. The evidence that can indicate the presence of azelaic acid in the structure of the produced nanofiber is the presence of a long peak at about 1698 cm^−1^, which indicates the presence of a dicarboxylic acid functional group, which can also be seen in the spectrum of azelaic acid. The peak in 2900–3000 cm^−1^is larger than the spectrum of azelaic acid powder, which indicates the presence of hydroxyl groups (O=H) in the nanofiber structure, which are the main purpose of creating transverse connections.

It should be noted that the present peak in 1093 cm^−1^, which is related to the presence of tension in the C=O ether bond, will be one of the important indicators in measuring the success rate in creating crosslinks.

And finally, the next spectrum that can be seen in Figure 6 is related to the nanofibers containing 70% of the drug on which the process of crosslinking has been done.

As it was mentioned before, the peak related to the C=O stretching of the ether at 1093 cm^−1^ and the peak related to the O=H stretching of the hydroxyl group at 2900–3000 cm^−1^ are very important in order to measure the success rate in creating crosslinks in polyvinyl alcohol so that the absorption ratio at 1093 cm^−1^ to absorption ratio at 2926 cm^−1^ is calculated for both spectra. If this ratio in the spectrum of the sample with transverse connections is higher than the sample without these connections, the process has been done correctly.

Here, this ratio is equal to 0.6 before establishing connections and then equal to 0.9, which indicates a relative success in establishing transverse connections, although the results of other analyses also confirm this issue.

### 3.6 Tensile Strength

Tensile test is one of the most common tests for measuring the mechanical properties of polymers. In this test, by pulling the sample via the jaws of the tension device, its reaction against applied forces can be determined. In other words, when the material is stretched, the Ultimate Tensile Strength (UTS) and Elongation at break (EB) of that material can be achieved. The output of this test is the stress–strain diagram, which is drawn based on the force applied for the elongation.

**FIGURE 7:**
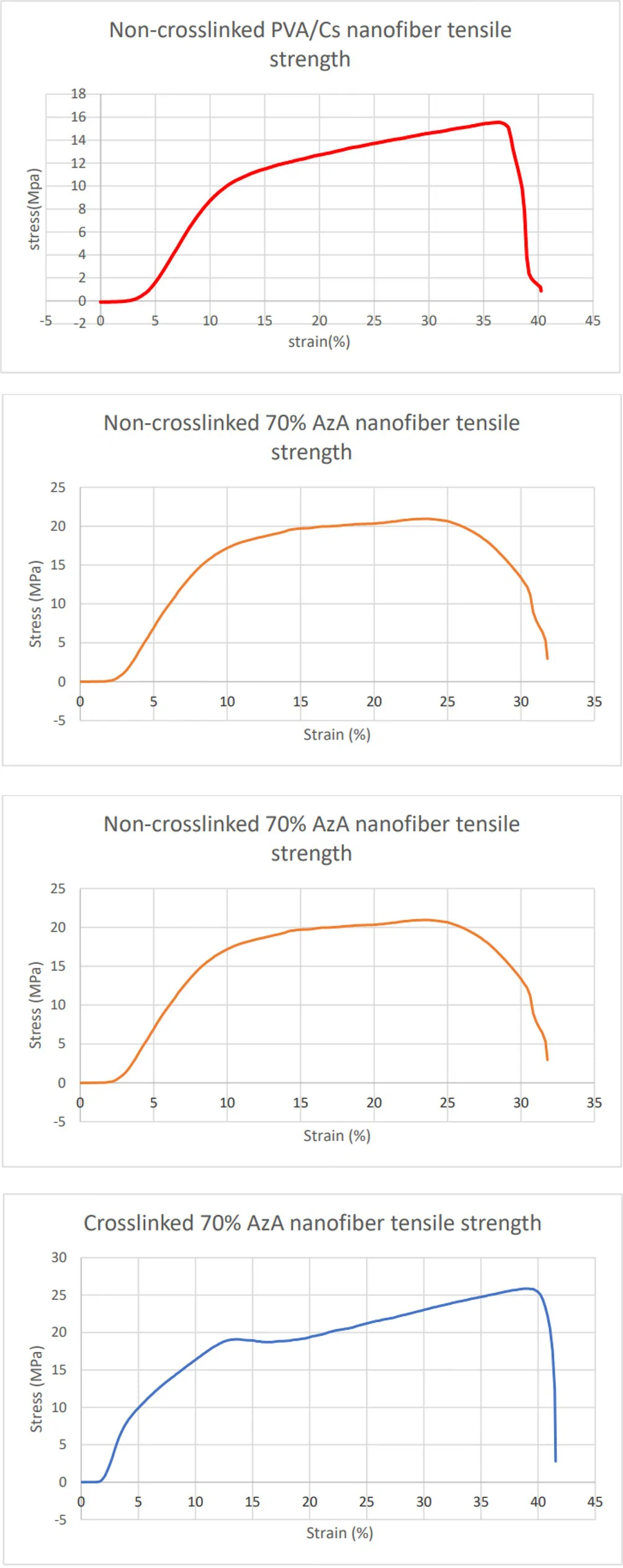
Stress–strain curves of blank and azelaic acid‐loaded nanofiber scaffolds before and after crosslinking. (A) Sample A: Blank nanofibers before crosslinking. (B) Sample B: Blank nanofibers after crosslinking. (C) Sample C: 70% azelaic acid‐loaded nanofibers before crosslinking. (D) Sample D: 70% azelaic acid‐loaded nanofibers after crosslinking.

The slope of the stress–strain curve in the linear region of the diagram is called Young's modulus. In this research, four samples of nanofibers were considered for tensile test, which include PVA/CS samples before and after cross‐linking and PVA/CS + AzA70% samples before and after cross‐linking (Table 5). The curves related to the mentioned nanofibers can be seen in Figure 7.

**TABLE 5:** Tensile properties of blank and azelaic acid‑loaded nanofiber scaffolds.

Sample code	Description	Maximum load (MPa)	Elongation at break (%)
A	Blank nanofiber before crosslinking	15.55	40.25
B	Blank nanofiber after crosslinking	41.20	26.00
C	Nanofiber with 70% azelaic acid before crosslinking	20.80	31.80
D	Nanofiber with 70% azelaic acid after crosslinking	25.85	41.00

Finally, according to the data obtained from this test, we came to the conclusion that adding azelaic acid to the nanofiber reduces its strength and increases its elongation.

### 3.7 Contact Angle Determination Test

**FIGURE 8:**
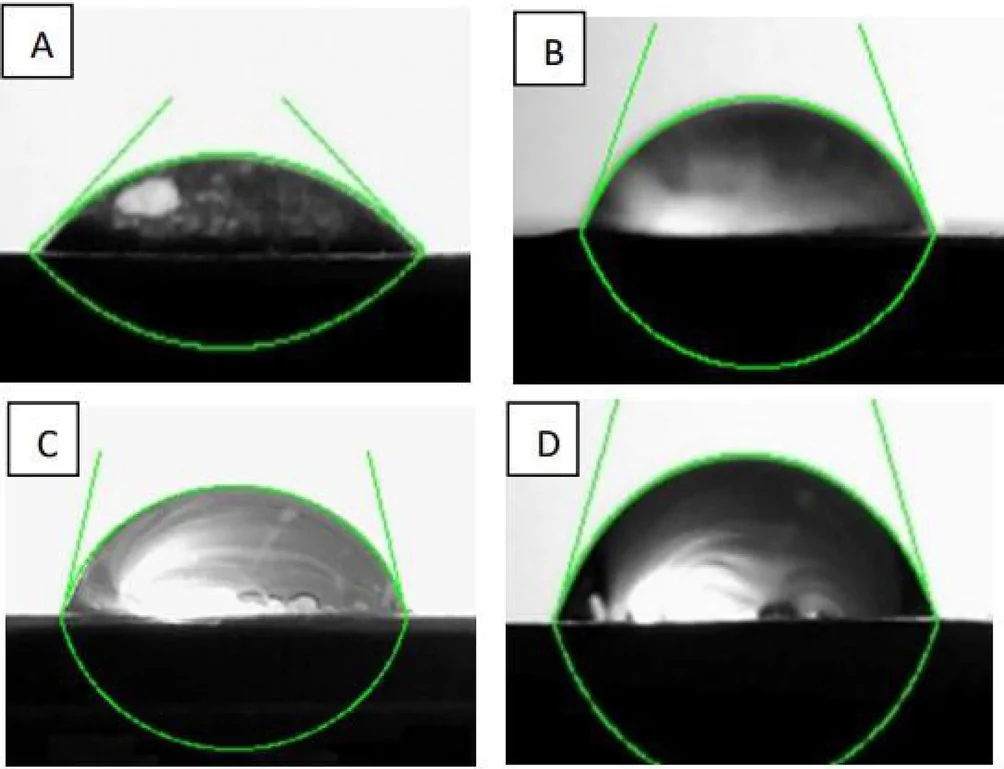
Images related to the contact angle of nanofibers. (A) Blank nanofiber before crosslinking; (B) Blank nanofiber after crosslinking; (C) Nanofiber with 70% azelaic acid before crosslinking; (D) Nanofiber with 70% azelaic acid after crosslinking.

Contact angle analysis is a test to measure the contact angle of a drop of water with a solid surface. Through this analysis, it is possible to measure the hydrophobicity and hydrophilicity of the desired sample. In general, contact angle is the angle that the last edge of a liquid makes with its underlying surface. In this study, the hydrophilicity of nanofiber was determined by measuring the contact angle of a drop of distilled water. Four samples of prepared nanofibers, including PVA/CS without drug before and after creating transverse connections and 70% AzA + PVA/CS before and after creating transverse connections, were considered for the contact angle test and their images are shown in Figure 8.

According to these images, cross‐linked nanofibers containing azelaic acid showed the highest contact angle and non‐cross‐linked PVA/CS nanofibers showed the lowest contact angle. Finally, it was seen that the addition of drug increased the contact angle of nanofibers compared to the sample without drug. The numbers related to the contact angle of the mentioned nanofibers are summarized in Table 6.

**TABLE 6:** Contact angle values of blank and azelaic acid‑loaded CS/PVA nanofiber scaffolds (mean ± SD).

Type of nanofiber	Contact angle (°), mean ± SD
CS/PVA	63.12 ± 2.3
Crosslinked CS/PVA	72.27 ± 5.3
CS/PVA + AzA 70%	67.28 ± 0.2
Crosslinked CS/PVA + AzA 70%	78.90 ± 6.1

### 3.8 Azelaic Acid Release

**FIGURE 9:**
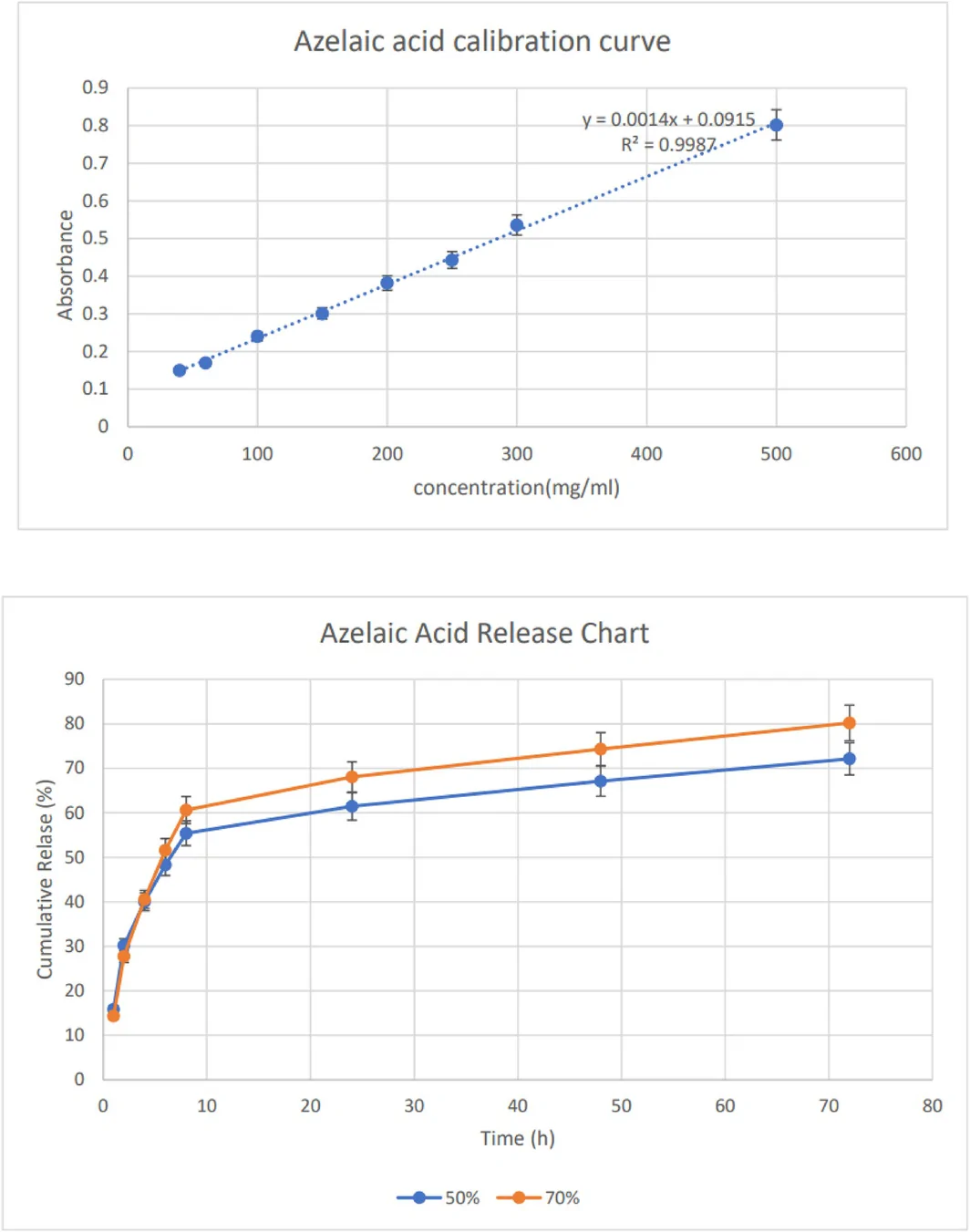
Calibration curve and in vitro release profiles of azelaic acid. (A) Calibration curve of azelaic acid in phosphate buffer. (B) In vitro release profiles from nanofiber scaffolds containing 50% and 70% azelaic acid.

In order to investigate the release of azelaic acid from nanofibers, after determining their absorption peak by UV spectrophotometer, its standard concentration‐absorption diagram at a wavelength of 203.8 nm for azelaic acid was drawn according to Figure 9.

Then the steps related to the release test were carried out. The release test was performed at neutral pH, and it is shown in Figure 9. According to the release pattern, most of the drug is rapidly released in the first 8 h. Then this release continues more slowly in the following hours. At neutral pH, between 50% and 60% of the azelaic acid is burst released during the first 8 h. After 72 h, this value increases to about 70%–80%.

### 3.9 Kinetic Modeling of Azelaic Acid Release From Nanofibers Bed

Several mathematical models were employed to understand the release mechanism of Azelaic acid from nanofibers bed. Correlation coefficient (R‐square) for different models including zero order and first order models, Higuchi and Korsmeyer‐peppas model were reported in Table 7. Obtained data from formulations containing 50% and 70% of drug were best fitted with Korsmeyer‐peppas model.

**TABLE 7:** Kinetic modeling of azelaic acid from nanofibers.

Model	Equation	*R* ^2^ Nanofibers containing 50% of drug	*R* ^2^ Nanofibers containing 70% of drug
Zero‐order	Mt = K0t	0.6346	0.6395
First‐order	Log Mt. = log M0 – K1 t/2.303	0.7726	0.8109
Higuchi	Mt/M∞ = KHt^1/2^	0.7867	0.7933
Korsmeyer‐peppas	Mt/M∞ = K′ t^n^ + b	0.8341	0.8385

### 3.10 Swelling Test

**FIGURE 10:**
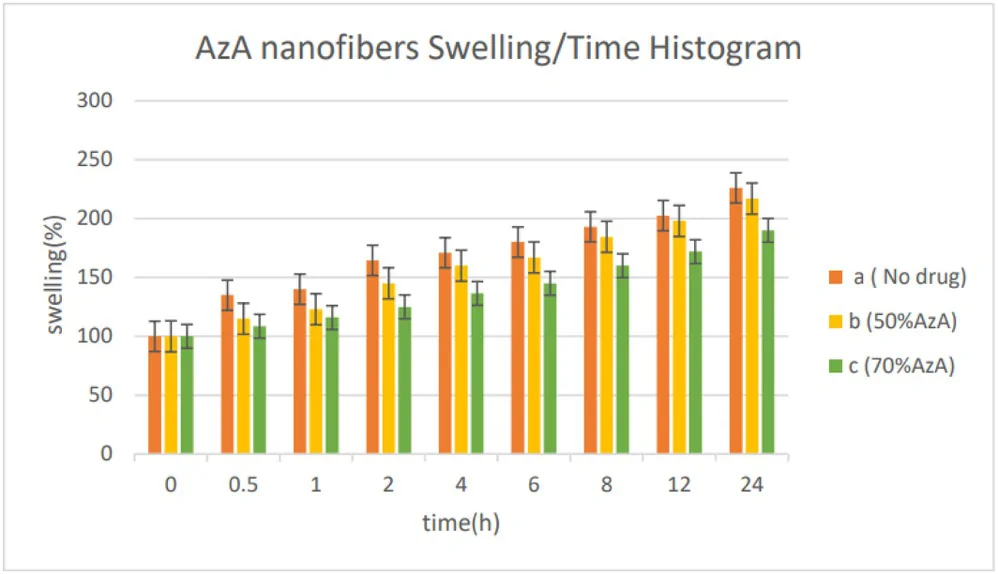
Swelling behavior of nanofiber scaffolds: Weight gain at different time points for blank and azelaic acid‐loaded formulations. (a) optimized nanofiber without drug, (b) optimized nanofiber containing 50% drug, (c) optimized nanofiber containing 70% drug.

The results obtained from the swelling test (percentage of sample's weight variations), which was performed on 3 nanofiber samples, are shown in Table 8 and Figure 10. In this table, sample A is a nanofiber sample without drugs and samples B and C have 50% and 70% of azelaic acid, respectively. These numbers are calculated according to Formula 1.

**TABLE 8:** Swelling behavior of nanofiber scaffolds in phosphate‐buffered saline at different time points (weight gain, %).

Sample code	0.5 h	1 h	2 h	4 h	6 h	8 h	12 h	24 h	Average weight gain (%)
A	135.0	140.0	164.5	171.0	180.0	193.0	202.5	226.0	176.5
B	115.0	123.0	145.4	160.0	167.0	184.5	198.0	213.0	163.0
C	108.5	116.0	125.0	136.5	145.0	160.0	172.0	190.0	144.0

Also, for a better comparison between the groups, the percentage of weight variations in different hours can be seen in Figure 10.

From the mentioned cases, it can be concluded that the presence of azelaic acid drug in the formulation structure, due to its lipophilic properties, can reduce the amount of water absorption by nanofibers.

### 3.11 Antibacterial Test

Determining the diameter of the inhibition zone or disk diffusion is a method to measure the antibacterial effects of formulations. In this method, bacteria are cultured in a plate and then the test discs are transferred to the plate, and after 1 day, the diameter of the area where the bacteria did not grow (inhibition zone) is measured. The bigger the inhibition zone, the stronger the antibacterial effect. The importance of the analysis to determine the diameter of the inhibition zone or disk diffusion has become much higher today due to the increase in the drug resistance of bacteria.

**FIGURE 11:**
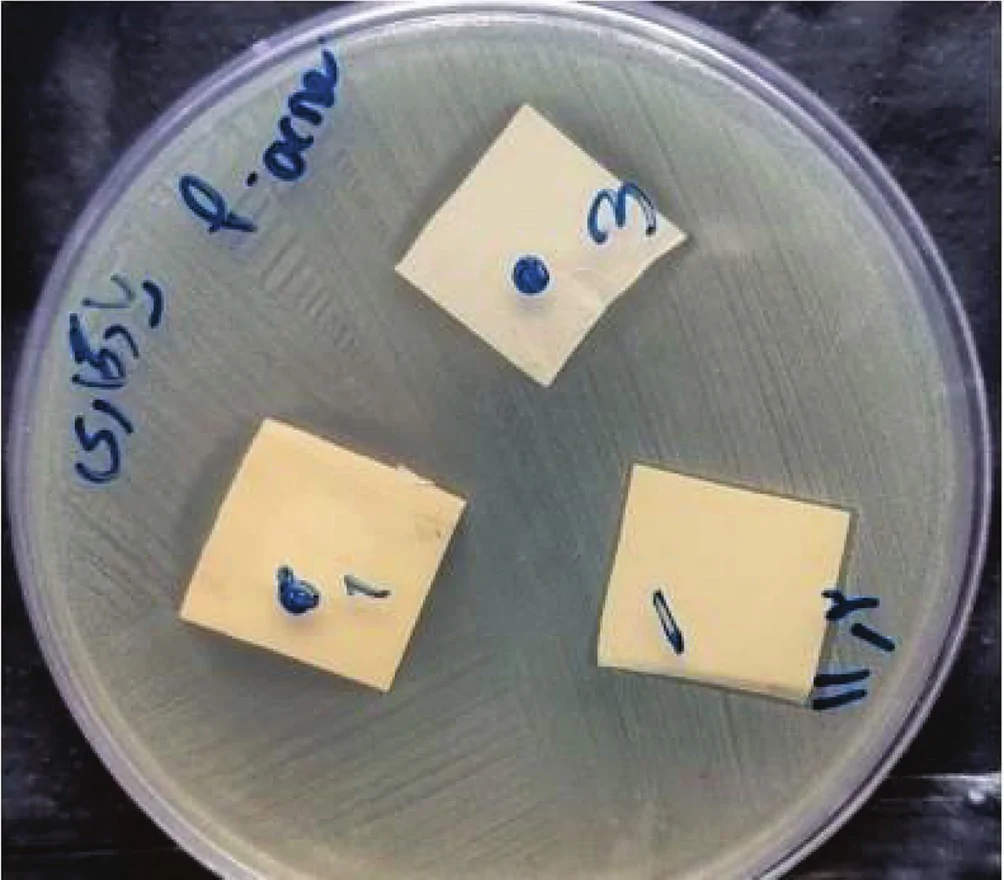
Inhibition zones of *C. acnes* produced by blank and azelaic acid‐loaded nanofiber scaffolds.

This test is performed for a wide range of gram positive and negative bacteria. *C. acnes* was used in this study. Three samples of prepared nanofibers including CS/PVA, CS/PVA + AzA50%, and CS/PVA + AzA70% were evaluated in this test after making transverse connections. The results can be seen in Figure 11 and Table 9. The azelaic acid‐loaded nanofiber scaffolds produced clear inhibition zones against *C. acnes*, whereas blank nanofibers did not show measurable antibacterial activity. These findings confirm that azelaic acid remains active after incorporation into the CS/PVA nanofiber matrix and can be released in amounts sufficient to inhibit bacterial growth under the test conditions.

**TABLE 9:** Antibacterial activity of nanofiber scaffolds against *C. acnes* (inhibition zone diameter).

Sample code	Type of nanofiber	Inhibition zone diameter against *C. acnes* (mm)
1	PVA/CS + AzA 70%	33
2	PVA/CS + AzA 50%	30
3	PVA/CS	No inhibition zone

### 3.12 Stability Analysis of Drug‐Containing Nanofibers

The physico‐chemical stability of the prepared nanofiber containing 70% azelaic acid was assessed for 12 weeks every 2 weeks. For performing stability analysis, the nanofibers were kept at a temperature of 45°C, and the changes in the organoleptic properties of the nanofibers were investigated as an indicator of physical stability, and the changes in the amount of drug in the nanofibers were investigated as an indicator of the chemical stability of the nanofibers.

During the investigations, no significant changes were observed in the organoleptic properties of nanofibers, including appearance, color, or smell, and therefore we conclude that the prepared nanofibers are physically stable.

To measure the amount of drug in the nanofiber for assessing the chemical stability, each time, 10 mg of prepared nanofiber was immersed in 5 mL of 96% ethanol for 1 h, and its optical absorption was obtained by UV spectrophotometer. The amount of drug in the solvent was calculated using the standard curve obtained for azelaic acid (Figure 9).

**FIGURE 12:**
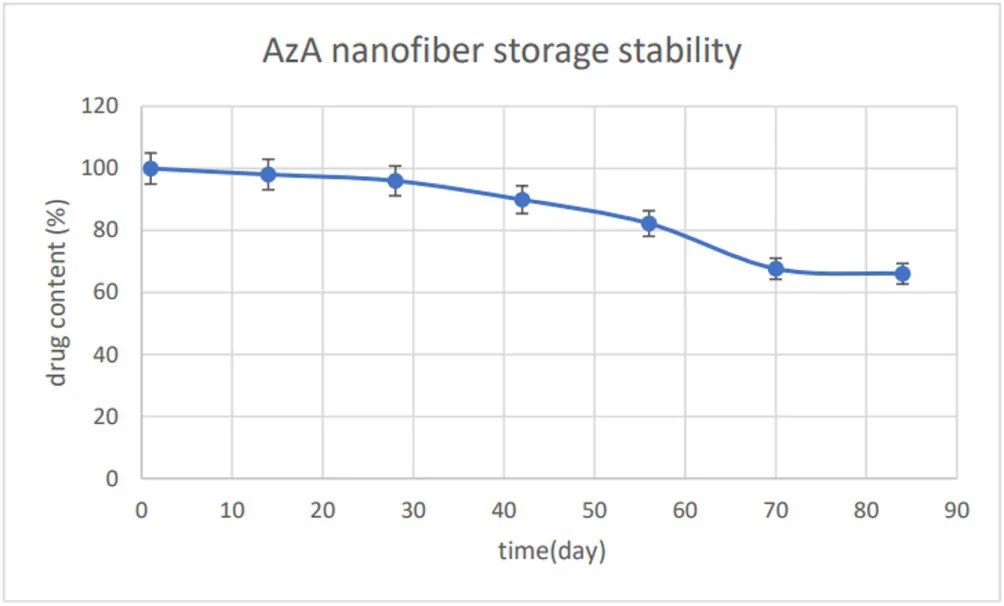
Chemical stability of azelaic acid‐loaded nanofiber scaffolds at 45°C over time.

The amount of drug obtained on day zero was considered as a reference, and then the data obtained during every 2 weeks were considered in proportion to the initial amount, and the percentage of the remaining drug in the nanofiber was calculated (Figure 12).

### 3.13 Statistical Analysis

Statistical analysis confirmed significant differences in nanofiber diameter (*p* = 0.009) and drug release profiles (*p* = 0.015) between the formulations (Table 10). A one‐way ANOVA test indicated a statistically significant impact of azelaic acid concentration on contact angle measurements (*p* = 0.022), suggesting that higher drug content increases hydrophobicity. Similarly, antibacterial effectiveness showed significant variation across formulations (*p* = 0.012). These findings validate the optimized formulation's effectiveness in drug delivery and antimicrobial activity.

**TABLE 10:** Statistical analysis of physicochemical and antibacterial outcomes.

Variable evaluated	Statistical test	*p*	Interpretation
Nanofiber diameter	Independent *t*‐test	0.009	Significant difference in fiber diameter across formulations
Drug release profile	Independent *t*‐test	0.015	Significant variation in drug release patterns among formulations
Contact angle	One‐way ANOVA	0.022	Azelaic acid concentration significantly increased hydrophobicity
Antibacterial effectiveness	One‐way ANOVA	0.012	Significant differences in antibacterial activity between formulations

## 4 Discussion

Acne vulgaris is a chronic inflammatory disease of the pilosebaceous units that causes the formation of comedones, papules, pustules, nodules, cysts, and scars. A wide range of topical treatments have been developed for acne, including topical solutions, gels, creams, and hydrogels [43, 44, 45, 46].

However, each of these dosage forms has its own disadvantages [44]. Based on the disadvantages of conventional topical formulations, several alternative novel formulations have been developed. Among these new methods, polymeric nanofiber patches (PNPs) have evolved as an alternative treatment for acne vulgaris due to their various advantages such as high surface‐to‐volume ratio, skin coverage, skin compatibility, high porosity, and ease of application. These properties cause nanofiber patches to be excellent candidates for many applications, including acne treatment, wound dressings, and drug delivery [13, 47, 48].

Several methods are available for manufacturing polymeric nanofibers. Among these methods, the electrospinning technique is the most common method due to its relative simplicity and scalability in the repeated production of nanofibers with dimensions of several micrometers to several nanometers [16].

In the present study, we successfully developed chitosan/polyvinyl alcohol (CS/PVA) electrospun nanofiber scaffolds loaded with azelaic acid as a topical platform for acne vulgaris and associated hyperpigmentation. While conventional topical dosage forms such as solutions, creams, and hydrogels are widely used in acne management, they may suffer from limited residence time on the skin, dosing inaccuracy, and cosmetic drawbacks such as visible residue or interference with skin oxygenation. By contrast, nanofiber‐based patches offer a high surface‐area‐to‐volume ratio, intimate contact with the skin, and the potential for controlled drug release, making them attractive candidates for dermatological applications including acne treatment [49, 50].

The CS/PVA system was selected to combine the biocompatibility, anti‐inflammatory potential, and structural similarity of chitosan to glycosaminoglycans with the favorable electrospinnability and mechanical properties of PVA. Using an optimized electrospinning process (20 kV applied voltage, 0.8 mL/h flow rate, 20 cm tip‐collector distance, 18 G needle, and 250 rpm collector speed), we were able to obtain bead‐free nanofibers with uniform morphology in both blank and azelaic acid‐loaded formulations. These formulation and process parameters were therefore considered suitable for the preparation of azelaic acid‐loaded nanofiber scaffolds intended for topical use [27, 51].

SEM analysis confirmed that all prepared nanofibers exhibited a uniform, bead‐free morphology, which is desirable for reproducible drug loading and mechanical performance. The average diameter of blank CS/PVA nanofibers was approximately 248 nm, with the majority of fibers distributed between 150 and 200 nm. Incorporation of azelaic acid led to a marked increase in fiber diameter, reaching mean values of 472 nm and 528 nm for formulations containing 50% and 70% drug, respectively. This diameter enlargement is consistent with the increased viscosity and altered surface tension of the spinning solution in the presence of high drug loading, which may limit the extent of jet stretching during electrospinning. Residual solvent entrapped in the fibers may also contribute to the observed increase in diameter. Overall, these morphological features indicate that high azelaic acid loading can be achieved without compromising the structural integrity of the nanofiber scaffold.

Mechanical testing revealed that the addition of azelaic acid reduced the tensile strength and stiffness of the nanofiber scaffolds compared with blank CS/PVA fibers. Although this decrease indicates that high drug loading may partly disrupt polymer–polymer interactions within the matrix, the optimized 70% azelaic acid formulation still retained sufficient strength to allow handling, application, and removal without tearing. For topical patches, excessive rigidity is not necessarily desirable, and a balance between mechanical robustness and flexibility is required to ensure good conformability to the skin surface. Our results suggest that the optimized formulation achieves such a compromise, although further in vivo handling assessments on human skin would be valuable [52, 53, 54, 55].

The wettability and swelling behavior of the nanofibers are critical for their interaction with the skin and for controlling drug release. Contact angle measurements showed that blank CS/PVA nanofibers were the most hydrophilic, consistent with the presence of abundant hydroxyl and amine groups, whereas incorporation of azelaic acid increased the contact angle and rendered the surface more hydrophobic. Formulations containing 50% drug displayed intermediate wettability, while those with 70% azelaic acid had the highest contact angle among the tested samples. Swelling experiments further demonstrated that the nanofibers were able to absorb and retain water up to approximately 1.5 times their dry weight, indicating a high capacity for hydration. Interestingly, the formulation with 70% azelaic acid exhibited the lowest water uptake, whereas the 50% formulation behaved similarly to the drug‐free scaffold. Taken together, these findings suggest that higher azelaic acid loading moderately reduces hydrophilicity and swelling, which may help to stabilize the scaffold structure in a moist environment while still providing sufficient moisture for comfortable skin adhesion.

Considering the combined data on morphology, mechanical behavior, wettability, and swelling, the formulation containing 70% azelaic acid appeared to provide the most favorable balance between drug loading capacity, structural integrity, and moisture stability, and was therefore selected as the optimized scaffold for further characterization.

In vitro release studies performed in phosphate‐buffered saline showed that nanofibers containing 50% azelaic acid released more than 70% of the loaded drug within 24 h, whereas the 70% formulation released approximately 80% over the same period. Both formulations exhibited an initial burst release phase during the first 8 h, followed by a slower, more sustained release up to 72 h. Such a biphasic release profile can be advantageous for acne therapy, as the early burst may rapidly achieve therapeutic concentrations at the skin surface, while the subsequent sustained phase may help maintain drug levels over a prolonged period without the need for frequent reapplication. Nevertheless, the relatively high initial release fraction also highlights the need for future in vivo studies to ensure that local concentrations remain within a well‐tolerated range for sensitive or compromised skin.

The antibacterial activity of the azelaic acid‐loaded nanofibers against *C. acnes* was confirmed by the presence of clear inhibition zones around the samples in agar diffusion assays. As expected, scaffolds with higher azelaic acid content generated larger inhibition zones than blank fibers, indicating that the released drug maintained its antimicrobial efficacy after the electrospinning and crosslinking processes. When compared qualitatively with reported minimum inhibitory concentration (MIC) values for azelaic acid against *C. acnes* and *Staphylococcus aureus*, our results suggest that the local drug levels achieved at the nanofiber–agar interface are sufficient to inhibit bacterial growth. However, because we did not include an azelaic acid solution control at equivalent drug doses, our current data do not allow us to conclude whether the nanofiber scaffold enhances, reduces, or does not alter the intrinsic antibacterial potency of azelaic acid. Future MIC and inhibition‐zone studies comparing nanofiber‐loaded azelaic acid with free azelaic acid are therefore warranted [8].

Finally, according to all the analyses and results obtained, it seems that nanofibers containing 70% (w/w) azelaic acid, in addition to better physicochemical stability than other samples, provide better drug release. Therefore, the nanofiber containing 70% azelaic acid was selected as the optimum formulation in this study. Although the 70% azelaic acid formulation demonstrated optimal physicochemical characteristics and antimicrobial properties, the potential side effects of high drug concentrations should be considered. Azelaic acid, at standard topical doses (15%–20%), is generally well‐tolerated; however, higher concentrations may increase the risk of skin irritation, erythema, dryness, or transient burning sensations. Previous studies suggest that prolonged exposure to high‐dose azelaic acid could potentially disrupt the skin barrier function or lead to hypersensitivity reactions in some individuals. Therefore, further in vivo studies and clinical trials are necessary to evaluate long‐term tolerability and safety.

Several clinical studies have reported mild to moderate adverse effects, especially with higher concentrations and prolonged use. In a multicenter open‐label trial by Dall'Oglio et al. [56], only one case of severe erythema was recorded among 44 patients using 15% AZA cream, and 90% of patients rated the tolerability as excellent [55]. Draelos et al. [57], in a Phase 3 randomized controlled trial involving 1156 patients, reported that drug‐related adverse events were slightly more common in the 15% AZA foam group (7.6%) compared to placebo (4.6%). These adverse effects—mostly burning, itching, and dryness—were localized to the application site and tended to peak within 4 weeks, resolving in over 96% of cases by the study's end [57]. Similarly, Tyring et al. [58] found in a parallel randomized controlled trial of 961 patients that the AZA foam group experienced a higher rate of drug‐related adverse effects (7.7%) compared to the control, with the most common being pain (3.5%), itching (1.3%), burning (1.4%), and dryness (1.0%). Despite these, overall tolerability was high, with 77.8% rating the treatment as excellent or good [58].

Taken together, these findings suggest that while AZA is largely well‐tolerated, localized skin irritation (erythema, burning, dryness, itching) remains the most common side effect. These are typically self‐limiting and may be mitigated by strategies such as gradual dose escalation, alternating application days, or use of moisturizers alongside treatment.

## 5 Conclusion

In this study, a polymeric nanofiber scaffold consisting of chitosan/polyvinyl alcohol loaded with azelaic acid was prepared using the electrospinning technique. The prepared nanofibers showed proper morphology and size and also had sufficient tensile strength. The nanofibers had medium hydrophilic properties; thus, they had the ability to exert a suitable hydrating effect on the skin and attach to it. The release pattern for azelaic acid was such that most of the drug was released in the first 8 h as a fast release; then this release continued in the following hours as a slow release. Nanofibers showed antibacterial effects against *C. acnes*, indicating that azelaic acid retains its activity after incorporation into the scaffold. These nanofiber carriers have the potential to improve topical delivery and local residence time of azelaic acid, although additional studies including appropriate positive controls are required to fully elucidate any contribution of the nanofiber matrix to antibacterial efficacy. The biodegradation and controlled release of drugs from these nanocarriers are crucial factors in achieving effective results in anti‐acne treatment. According to these advantages, azelaic acid‐loaded nanofibers may serve as a promising alternative to conventional therapies for the treatment of acne and skin hyperpigmentation.

## Author Contributions

All authors contributed equally in different parts of this study including conception or design of the work, analysis, interpretation of data, drafting the work, or revising it critically for important intellectual content, and final approval of the version to be published.

## Funding

This work was supported by Tehran University of Medical Sciences and Health Services, 9613120040, 2019.

## Conflicts of Interest

The authors declare no conflicts of interest.

## Data Availability

The data that support the findings of this study are available on request from the corresponding author. The data are not publicly available due to privacy or ethical restrictions.
